# The Human Acid-Sensing Ion Channel ASIC1a: Evidence for a Homotetrameric Assembly State at the Cell Surface

**DOI:** 10.1371/journal.pone.0135191

**Published:** 2015-08-07

**Authors:** Miguel Xavier van Bemmelen, Delphine Huser, Ivan Gautschi, Laurent Schild

**Affiliations:** Department of Pharmacology & Toxicology, Faculty of Biology and Medicine, Lausanne University, Lausanne, Switzerland; University of South Alabama, UNITED STATES

## Abstract

The chicken acid-sensing ion channel ASIC1 has been crystallized as a homotrimer. We address here the oligomeric state of the functional ASIC1 in situ at the cell surface. The oligomeric states of functional ASIC1a and mutants with additional cysteines introduced in the extracellular pore vestibule were resolved on SDS-PAGE. The functional ASIC1 complexes were stabilized at the cell surface of *Xenopus laevis* oocytes or CHO cells either using the sulfhydryl crosslinker BMOE, or sodium tetrathionate (NaTT). Under these different crosslinking conditions ASIC1a migrates as four distinct oligomeric states that correspond by mass to multiples of a single ASIC1a subunit. The relative importance of each of the four ASIC1a oligomers was critically dependent on the availability of cysteines in the transmembrane domain for crosslinking, consistent with the presence of ASIC1a homo-oligomers. The expression of ASIC1a monomers, trimeric or tetrameric concatemeric cDNA constructs resulted in functional channels. The resulting ASIC1a complexes are resolved as a predominant tetramer over the other oligomeric forms, after stabilization with BMOE or NaTT and SDS-PAGE/western blot analysis. Our data identify a major ASIC1a homotetramer at the surface membrane of the cell expressing functional ASIC1a channel.

## Introduction

Acid-sensing ion channels (ASICs) are proton-gated cation channels that belong to the ENaC/degenerin family of non-voltage-gated sodium channels. This family, includes in vertebrates the epithelial sodium channel ENaC, and the ASICs subfamilies. Six ASIC proteins have been identified (ASIC1a, ASIC1b, ASIC2a, ASIC2b, ASIC3 and ASIC4) that assemble as homo or heteromers to form voltage-insensitive channels permeable to Na^+^ and Ca^2+^. ASICs are localized in cell bodies, in dendrites and in post-synaptic dendritic spines, suggesting a role in synaptic transmission. ASIC1 knockout mice show decreased acid-induced inward currents and reduced long-term potentiation associated either with fear-related behaviors, altered learning and memory processes, or pain sensation [1]. The pH-dependence of ASIC1 at physiological pH values is characterized by a channel desensitization upon acidification of the extracellular milieu between pH7.4 and 7.0; the channel opens transiently when the pH drops below 7.0 [2].

Chicken ASIC1 (cASIC1) has been crystalized as a homotrimer [3, 4]. Each subunit comprises a large extracellular domain located between two transmembrane α helices (TM1 and TM2) arranged pseudosymmetrically around a closed pore. This conformation was interpreted as the desensitized state of the channel. The TM2 of each subunit is located close to the three-fold axis, lining the putative ion channel pore, while the TM1 helices lie at the periphery where they probably establish most of the contact with the lipid bilayer. The crossing of the TM2 helices identifies an extended physical gate in the transmembrane domain that precludes the flow of ions between the extracellular and the intracellular milieus. The peptide Psalmotoxin 1 (PcTx1) in the venom of the tarantula *Psalmopoeus cambridgei* inhibits homomeric ASIC1a channels at nanomolar concentrations [5]. A crystal structure of cASIC1 bound to psalmotoxin-1 obtained at two different proton concentrations shows a rearrangement both of the inter-subunit interactions at the extracellular domain and of the transmembrane α helices [6]. Even though cASIC1 was crystalized as homotrimers in different channel conformations, there is presently only little evidence showing that any of these high resolution structures represent the functional channel in situ.

The state of oligomerization at the cell membrane of the members of the ENaC/degenerin channel family has been quite controversial. Functional and biochemical studies on ENaC supported a tetrameric subunit organization [7–10]. Studies using fluorescence microscopy proposed an ENaC oligomeric state consistent with a previously reported 9 subunits stoichiometry [11, 12]. A recent study using single-molecule imaging also found evidence for a trimeric subunit composition of ASIC1 on the cell surface [13]. These discrepancies on the nature of the subunit organization of the functional ASIC or ENaC channels can be attributed to the absence of a single reliable technique allowing a definitive answer to this question.

In the present report we have addressed the state of subunit oligomerization of the ASIC1a channel complex at the cell surface using a classical biochemical approach. We observed that functional ASIC1a channels consistently migrate on SDS-PAGE as four distinct oligomers that represent monomers, dimers, trimers and tetramers of ASIC1a subunits, the latter being the most abundant oligomer.

## Results

**Fig 1:**
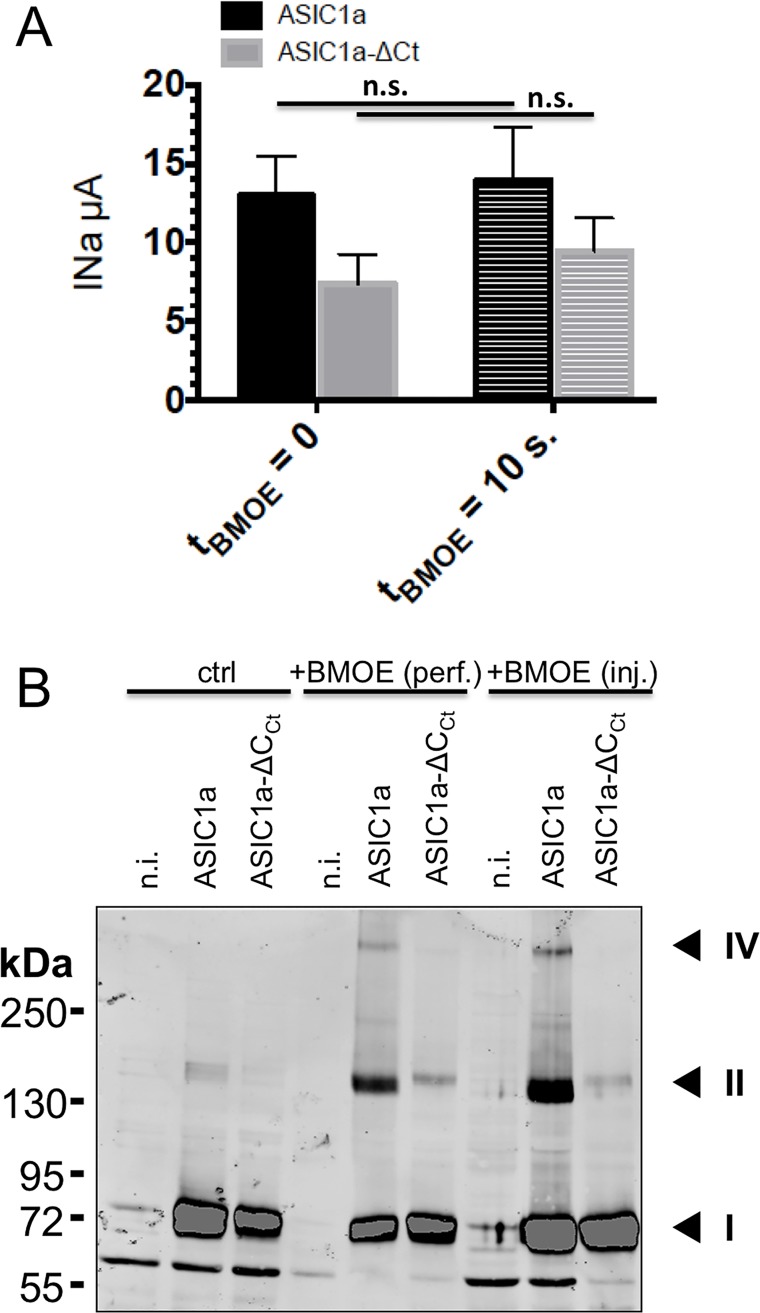
Effects of intracellularly applied BMOE on hASIC1a activity and oligomerization. A: Currents were recorded in cut-open oocytes expressing either wild type ASIC1a (black bars n = 18) or ASIC1a-ΔC_Ct_ lacking cysteines in the C-terminus (grey bars, n = 17) before and after 10 s of intracellular perfusion with 2 mM BMOE (+BMOE). Bars represent mean ± SE. B: Anti-ASIC1a western from oocytes, either non-injected (n.i.), or expressing ASIC1a or ASIC1a-ΔC_Ct_, untreated (left), or treated with 2 mM BMOE by internally perfusion (perf.) or intracellular injection (inj.). Numbers I, II, and IV designate the most prominent bands that are specific for ASIC1a, II and IV having apparent weight sizes that are, respectively, twice and four times that of I.

Our strategy consisted in the isolation of ASIC1a channel complexes in situ, and the analysis of the ASIC1a oligomers resolved by SDS-PAGE. We stabilized the ASIC1a complex according to previous observations that, under oxidant conditions, intracellular cysteine residues at the C-terminus of ASIC1a participate in the formation of intersubunit disulfide bonds [14]. In the experiments shown in Fig 1, we used the membrane-permeant BMOE, a short-arm (8 Å) maleimide crosslinker for covalent, irreversible, DDT-resistant conjugation of pairs of sulfhydryl groups of both the wild type human ASIC1a and a mutant lacking the C-terminal cysteines (ASIC1a-ΔC_Ct_) expressed in Xenopus oocytes; the stabilized oligomerization states were then resolved on SDS-gel. *In situ* crosslinking by intracellular application of BMOE (2mM) to ASIC1a wt or ASIC1a-ΔC_Ct_ did not affect channel activity as shown by the magnitude of the current elicited by pH 5.5 (Fig 1A). The SDS-PAGE/western blot made under reducing conditions in Fig 1B from oocytes with intracellular application of BMOE, revealed two high molecular weight (MW) bands that were absent or considerably reduced for the cysteine-free ASIC1 mutant in the C-terminus (ASIC1a-ΔC_Ct_). We have identified a ASIC1 protein on SDS-gel that migrates more slowly than the 250kDa MW marker and therefore expected to have a molecular mass higher than 250 kDa. We have estimated the apparent MW values of the oligomers identified as distinct bands on blots of samples treated with or without BMOE as indicated in Methods, and obtained the following values (mean±SD, n = 7): 72±2, 156±7, and 329±19 kDa for ASIC1a wt and 73±2 and 164±6 kDa for ASIC1a-ΔC_Ct_. The estimated molecular weights of these proteins detected in anti-ASIC1 western blots correspond to that expected for ASIC1a monomers (band I), dimers (band II) and dimers of dimers (band IV), consistent with the results of Zha et al. obtained with ASIC1a treated with H_2_O_2_ [14].

**Fig 2:**
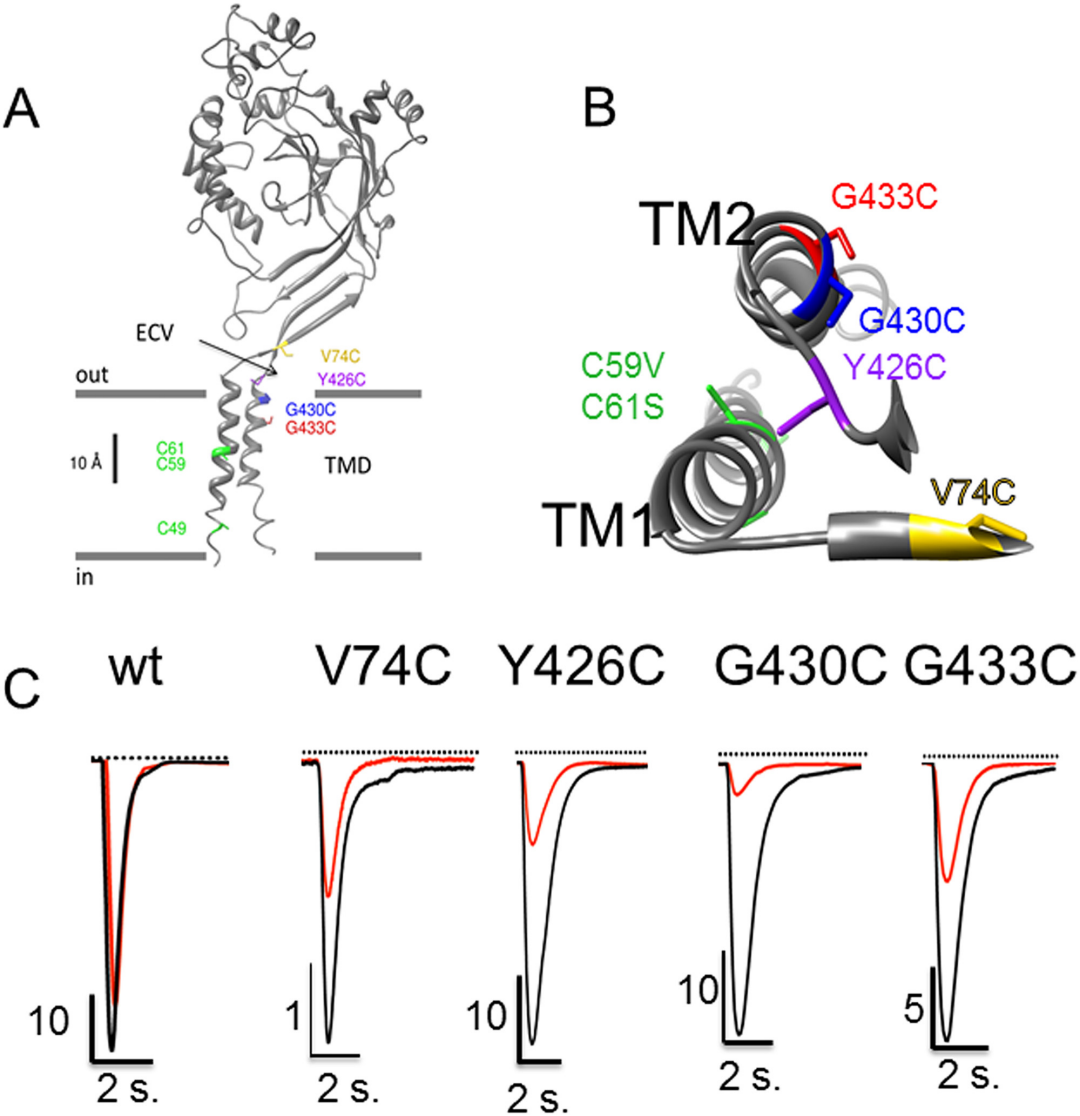
Native cysteines and cysteine substitutions in the transmembrane domain of ASIC1a. A: Model structure of a human ASIC1a subunit based on the chicken ASIC1 crystal structure as published elsewhere [4]: the subunit is made of 2 transmembrane α helices (TM1 and TM2); the cysteines C49, C59 and C61 in the transmembrane α helix 1 (TM1) are shown in green. The substituted cysteines G430C and G433C are in the TM2, and V74C and Y426C are located at the entrance of the channel pore in the extracellular vestibule (ECV). B: Top view of the transmembrane α helices TM1 and TM2 of a single ASIC1a subunit, with the pore lining residues G433C and G430C according to Li et al. 2011. C: Representative recordings of ASIC1a currents elicited at pH 5.5 in the absence (black) or in the presence (red) of 100 μM Cd^2+^ in xenopus oocytes expressing either ASIC1a wt or the V74C, Y426C, G430C, G433C mutants.

From these experiments, we hypothesized that crosslinking of the cysteines in the C-terminus favors the stabilization of ASIC1a homodimers, and eventually dimers of dimers migrating as a band higher than 250 kDa. We tested whether different combinations of cysteine deletions and substitutions would differentially affect the degree of BMOE, as resolved by different ASIC1a oligomers on SDS-gels. We identified residues G430 and G433 in the TM2 α helix of ASIC1a, Y426 in the short loop preceding the TM2, and V74 in the prolongation of the external end of the TM1 (Fig 2A) [15] as candidates for cysteine substitution and crosslinking by BMOE. The V74C, Y426C, G433C, and G430C individual substitutions were generated in the ASIC1a-ΔC_Ct_ background lacking the C-terminal cysteines. We verified that these ASIC1 mutants were functional (Fig 2B) and retain the functional and pharmacological characteristics of ASIC1a wt (Table 1); we observed that these cysteine substitutions confer a channel block by extracellular Cd^2+^. Full activity of the V74C mutant could be restored upon extracellular treatment with DTT, while BMOE drastically and irreversibly decreased the activity of both the Y426C and G430C (Table 1). This is consistent with cysteines located in the pore vestibule of the channel and accessible to extracellular ligands and/or potentially involved in disulfide interactions.

**Table 1:** Functional characteristics of the ASIC1a constructs.

	wt	ΔC_Ct_	V74C	Y426C	G430C	G433C
I_Na_ (μA)	55.33±26.88	45.06±20.6	2.9±2.4**	22.65±14.22	22.75±14.73	3.6±3.8 **
pH_0.5_	6.35±0.02	6.35±0.014	6.36±0.04	6.57±0.01	6.36±0.01	6.55±0.02
IC_50_ (μM) amiloride	219±43	150±24	7.1±1.4	31.9±4.2	35.3±4.9	2.5±0.51
IC_50_ Cd^2+^ (mM)	0.527±0.049	1.16±0.095	< 0,01	0.032±0.0016*	0.010±0.001*	0.057±0.0037*
I_Na_ + BMOE (% of control)	ND	90.2±10.0	115.7±24	6.4±0.18**	9.5±0.17**	89.1±13.3
I_Na_ (μA) +DTT	ND	38.04±15.24	25.84±15.72	29.92±16.8	26.29±19.37	3.79±2.92

ASIC1a currents (I_Na_) were elicited at pH 6.0. ΔC_Ct_ denotes ASIC1a mutant lacking the cysteines in the C-terminus. BMOE (2mM), Cd^2+^ and DTT (10 mM) were added in the extracellular medium.

** denotes statistical significance p<0.01 and

* p<0.05, unpaired *t*-test for the mutant compared to ASIC1a wt. denotes p<0.01 for treated versus untreated condition.

Current values (μA or % of control) are expressed as mean ± SD

IC_0.5_ and pH_0.5_ are expressed as best-fit values ± 95% confidence intervals of at least 4 independent experiments

**Fig 3:**
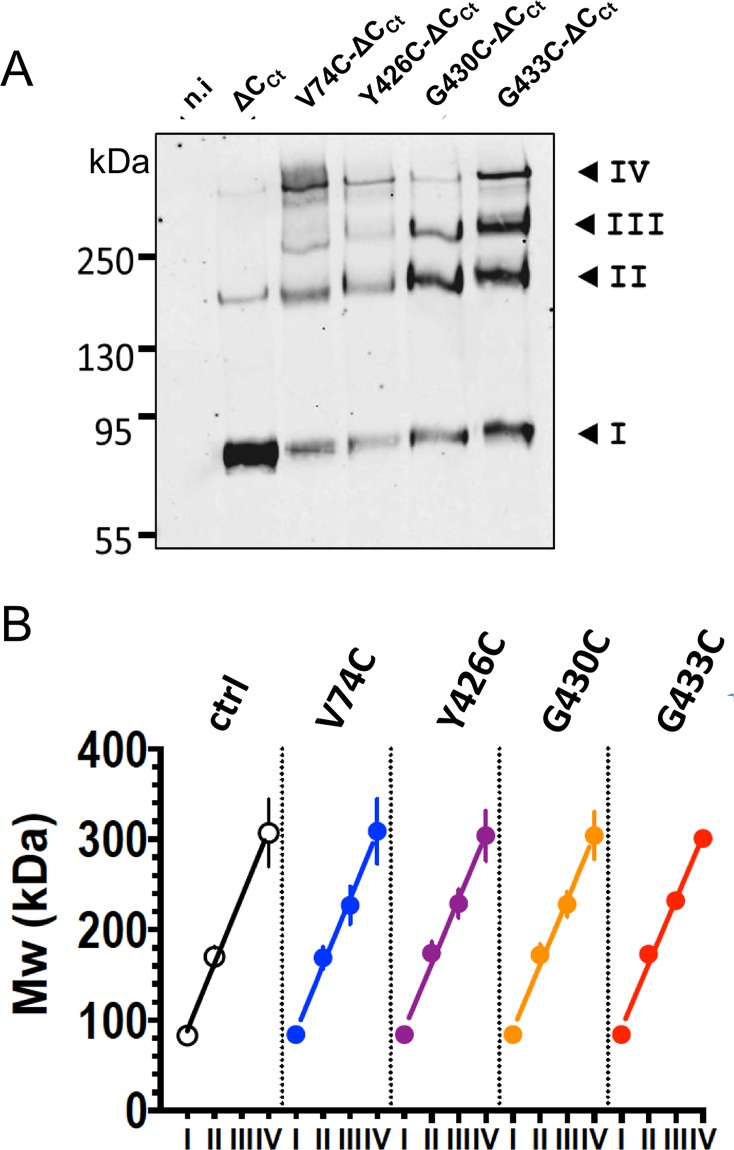
ASIC1 oligomeric states resolved by SDS-PAGE after crosslinking with BMOE. A: Anti-Anti-His-tag western blot of the surface-biotinylated protein fractions from either non-injected oocytes (n.i.), or oocytes expressing either the His_8_-tagged form of ASIC1a-ΔC_Ct_ (ΔC_Ct_), lacking cysteines in the C-terminus, or the corresponding cysteine-substitution mutants V74C-ΔC_Ct_, Y426C-ΔC_Ct_, G430C-ΔC_Ct_, and G433C-ΔC_Ct_. Crosslinking with 2 mM BMOE was performed at the cell surface; the cell-surface biotinylated proteins were affinity-purified on streptavidin beads. Numbers I to IV, designate the four most prominent bands. B: Apparent Mw values of the ASIC1 oligomers (kDa, mean ±SD) estimated for each of the four main bands (I to IV) for the different constructs, as in A. Lines represents linear regression of the average values, with slopes ranging from 71 ± 4.2 to 73.8 ± 4.6 kDa for the different ASIC1a cDNA constructs.

The oligomeric states of ASIC1a mutants in the ΔC_Ct_ background ASIC1a-ΔC_Ct_, V74C-ΔC_Ct_, Y426C-ΔC_Ct_, G430C-ΔC_Ct_, G433C-ΔC_Ct_, after treatment with BMOE and cell-surface biotinylation of intact oocytes, were resolved by SDS-PAGE analysis under reducing conditions. Western blot analysis of fractions bound to streptavidin beads shows that ASIC1a-ΔC_Ct_ runs on SDS-PAGE as a major band corresponding to the mass of a monomer (band I), and as a weaker ~160 kDa band (band II) consistent with an ASIC1a dimer (Fig 3A). Each of the BMOE-treated mutants, V74C-ΔC_Ct_, Y426C-C_Ct_, G430C-ΔC_Ct_ or G433C-ΔC_Ct_, runs as a ladder of four distinct bands (I to IV) with similar migration patterns. When compared to ASIC1a-ΔC_Ct_, the increase in intensity of the three upper bands (II, III, and IV) for the cysteine mutants correlates with a decrease in the amount of monomers. As shown in Fig 3B, the apparent MW (n = 4) of each band I to IV increases linearly for all the ASIC1a-ΔC_Ct_ constructs, with an average slope of 72±4 kDa that corresponds to the expected mass of a single ASIC1a subunit. Together these experiments support the assembly of ASIC1a as a tetramer at the surface of oocytes expressing functional channels.

**Fig 4:**
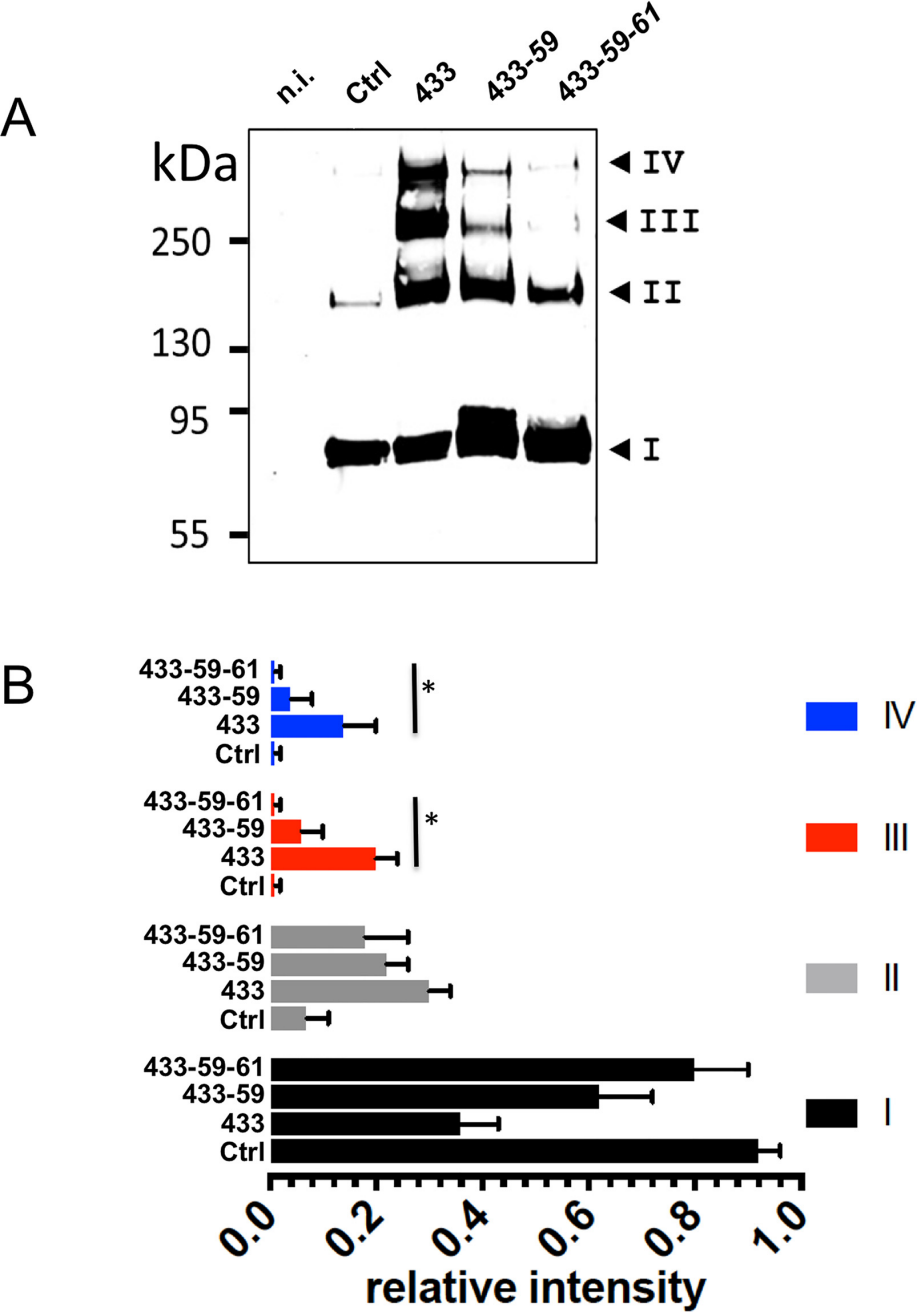
Contribution of cysteines C59 and C61 in the first transmembrane helix (TM1) to the stabilization of the ASIC1a complex by BMOE. A: ASIC1a oligomeric states, resolved by SDS-PAGE under reducing conditions and detected by anti-His-tag western blotting (same experimental procedure as in Fig 3A), of the His_8_-tagged forms of ASIC1a-ΔC_Ct_ as control (Ctrl), and the cysteine substitution mutant G433C-ΔC_Ct_ (433), G433C-C59V-ΔC_Ct_ (433–59) or G433C-C59V-C61S-ΔC_Ct_ (433-59-61). Numbers I to IV have the same meaning as in Fig 3. B: Relative intensities (mean ±SEM, n = 4) of each of the 4 bands (I to IV) for ASIC1a identified on SDS-PAGE from cells expressing ASIC1a-ΔC_Ct_, (C), G433C-ΔC_Ct_ (433) G433C-C59V-ΔC_Ct_ (433–59) and G433C-C59V-C61S-ΔC_Ct_ (433-59-61). The average molecular weight estimated for the four bands I, II, III, IV are respectively 80±10, 160±6, 230±9 and 300±19 kDa (Mw±SD, n = 4) for the G433C-ΔC_Ct_, G433C-C59V-ΔC_Ct_, and G433C-C59V-C61S-ΔC_Ct_ constructs. * denotes p<0.01

According to its homo-bifunctional nature, BMOE is expected to stabilize only dimers of ASIC1a-ΔC_Ct_, provided that BMOE crosslinks exclusively the cysteine residues introduced in the extracellular vestibule on each subunit. Since we used mutants lacking the C-terminal cysteines, the stabilization of oligomers of higher order than dimers observed in Fig 3A requires the participation of additional native cysteines. Likely candidates are cysteines C59 and C61 in the TM1 located at a crosslinking distance from the engineered cysteines for BMOE (see Fig 2B). Fig 4A compares the oligomerization patterns on SDS-PAGE of ASIC1a-ΔC_Ct_ (Ctrl), G433C-ΔC_Ct_ (433), G433C-C59V-ΔC_Ct_ (433–*59*), and G433C-C59V-C61S-ΔC_Ct_ (433-*59-61*) after ASIC1a crosslinking with BMOE at the cell surface. Crosslinking of G433C-ΔC_Ct_ at the cell surface with BMOE yields four distinct bands consistent with data on Fig 3A. The two high MW G433C-ΔC_Ct_ oligomers corresponding to bands III and IV (234±8 and 303±17 kDa, n = 4), were considerably less abundant for the mutant carrying the C59V (G433C-C59V-ΔC_Ct_) and almost disappeared for the double C59V-C61S substitution mutant (G433C-C59V-C61S-ΔC_Ct_). Fig 4B illustrates the relative intensity of each channel oligomer migrating as bands I to IV, for the ASIC1a-ΔC_Ct_ and the three cysteine mutants. ASIC1a-ΔC_Ct_ (Ctrl) migrates essentially as a monomer, and less than 10% of ASIC1a migrates as band II. In contrast, the intensities of bands I, II, III, and IV of BMOE-treated G433C-ΔC_Ct_ (433) are very similar. The substitution mutants C59V (433–59) and C59V-C61S (433-59-61) show respectively a decrease and the almost disappearance of bands III and IV. Thus, the C59V and C61S substitutions reverse the effect of G433C on the stabilization by BMOE of ASIC1a-ΔC_Ct_ complex. The resolution of high MW ASIC1a-ΔC_Ct_ oligomers on SDS-PAGE critically that depend on the availability of G433C, C59 and C61 for crosslinking by BMOE, supports the notion that these oligomers are homomultimers made of ASIC1a subunits.

**Fig 5:**
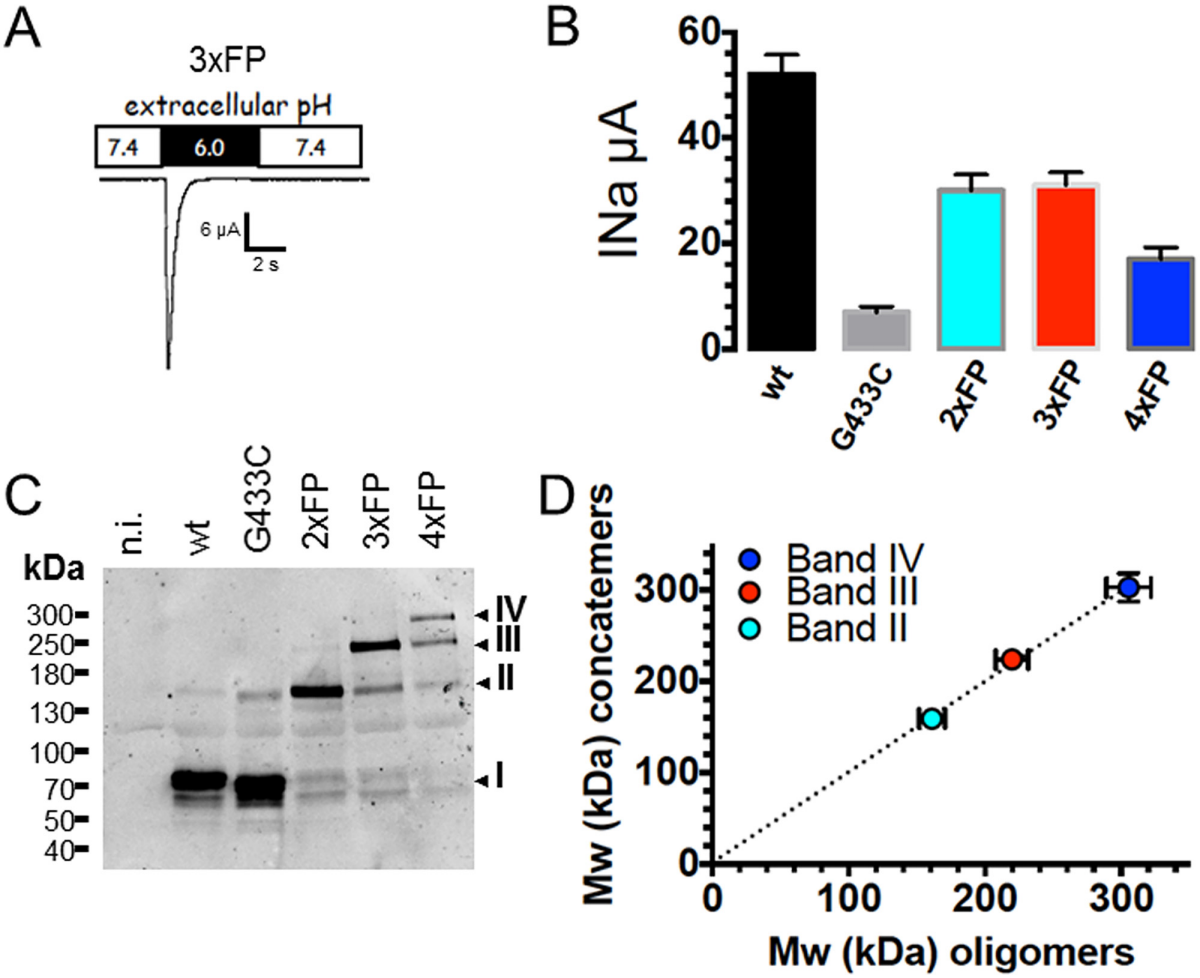
Functional expression of concatenated ASIC1a fusion proteins. A: Representative recording of an ASIC1a current in Xenopus oocytes expressing a fusion protein made of three ASIC1a subunit proteins, each with intact C-termini, and linked in a head to tail fashion (3xFP). B: Average (mean ± SD) of ASIC1a currents elicited at pH 6.0 measured in Xenopus oocytes expressing monomeric hASIC1a (wt or G433C mutant) or fusion proteins made of two (2xFP), three (3xFP), or four (4xFP) ASIC1a subunit proteins. All constructs comprise a single, N-terminal, His_8_ tag. C: Anti-ASIC1a western blot obtained from oocytes expressing the same ASIC1a constructs as in B; I, II, III, IV have the same meaning as in Fig 3A. D: Relationship between the molecular weights of bands I, II, III, IV estimated from ASIC1a oligomers crosslinked with BMOE (as in Fig 3A) and the size (kDa) of dimeric (gray), trimeric (red) and tetrameric (blue) ASIC1a fusion proteins; the straight dotted line represents the linear regression fit forced to the axes origin; the slope of the regression line is 0.993 ± 0.052.

To provide further evidence that the four ASIC1a oligomers identified as bands I, II, III, and IV on Figs 3A and 4A represent ASIC1a homomultimers we verified that their migration patterns on SDS-gels are similar with those of fusion proteins made of 2, 3 or 4 concatenated ASIC1a subunits (2_ASIC_FP, 3_ASIC_FP, 4_ASIC_FP) assembled in a head to tail fashion. These fusion proteins were functional when expressed in Xenopus oocytes and generated typical ASIC1 currents (Fig 5A and 5B) with similar sensitivities to activation by protons, and with a conserved sensitivity to block by amiloride (Table 2). The blot of Fig 5C shows that 2_ASIC_FP, 3_ASIC_FP, 4_ASIC_FP migrate as bands II, III, and IV respectively, with apparent MWs expected for concatemers made of 2, 3, or 4 ASIC1a subunits. Additional bands of lower MWs were detected, corresponding by mass to single or multiples of ASIC1a subunits, suggesting a cleavage or an incomplete biosynthesis of the concatemeric fusion proteins. Fig 5D shows the correlation (slope of 0.992 ± 0.052) between the estimated MW of bands II, III, and IV of ASIC1a oligomers crosslinked with BMOE as in Fig 3 (X axis), and the MW of bands corresponding to full-length 2_ASIC_FP, 3_ASIC_FP, 4_ASIC_FP concatemers (Y axis). This strict correlation supports that the oligomers migrating as bands I, II, III, IV represent respectively monomers, homodimers, -trimers, and -tetramers of ASIC1a.

**Table 2:** 

	2_ASIC_FP	3_ASIC_FP	4_ASIC_FP
**pH**_**0.5**_	**6.35±0.02**	**6.45±0.02**	**6.25±0.02**
**IC**_**50**_ **(μM) amiloride**	**21.4±4.1**	**100±16.5**	**37.4±7.5**

Values are expressed as best-fit values ± 95% confidence intervals of at least 4 independent experiments

**Fig 6:**
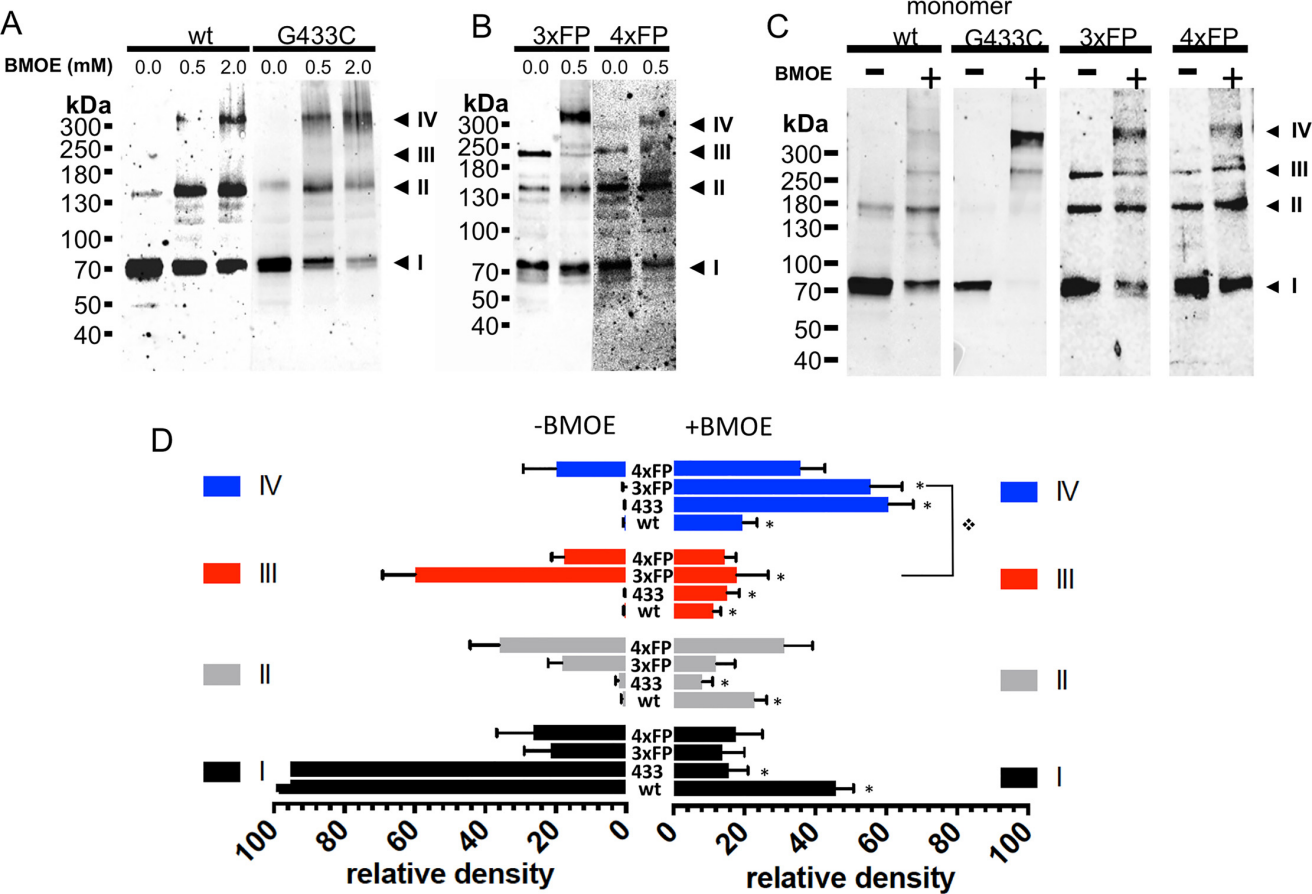
Oligomeric states of ASIC1a trimeric and tetrameric fusion proteins at the surface of cells expressing functional ASIC1a. A-B: Anti-ASIC1a western blot of surface biotinylated fractions of oocytes, treated with vehicle or with BMOE (0.5 or 2 mM) before lysis, expressing either ASIC1a wt or G433C (A) or 3_ASIC_FP (3xFP) or 4_ASIC_FP (4xFP) (B). C: Anti-ASIC1a western blot of ASIC1a in cell-surface biotinylated fractions of CHO cells expressing monomeric ASIC1a wt, or G433C mutant, 3xFP, or 4xFP fusion proteins with or without treatment with BMOE before lysis. I, II, III, IV have the same meaning as in previous figures. D: Relative intensities (mean ±SD) of each of the 4 bands (I to IV) ASIC1a oligomers from cell-surface biotinylated fractions of *Xenopus* oocytes and CHO cells expressing ASIC1a (wt, n = 17), or G433C (433, n = 17) monomeric forms, or 3xFP (n = 8), or 4xFP (n = 8) fusion proteins treated with either vehicle or 0.5 mM BMOE. Symbol * denotes p<0.01 for comparison between condition -BMOE and +BMOE, ❖ p<0.01 for the indicated comparison.

We have observed that the expression of trimeric or tetrameric fusion proteins generates both typical ASIC1a currents and thus functional channels (see Fig 5a and 5b). It is however quite unlikely that both the trimeric and tetrameric ASIC1a assembly states generate functional channels at the cell surface. Different hypothesis can be proposed: first, the functional ASIC1a is a trimer and only three subunits of the 4_ASIC_FP participate in the formation of the functional channel. Alternatively, the 3_ASIC_FP concatemer associates with a single subunit available in the cell to form a functional tetramer at the cell surface. In other words, the question is whether the 3_ASIC_FP proteins needs to be complemented to form a functional ASIC1a at the cell surface. The biotinylated fractions of monomeric ASIC1a or G433C (with intact C-terminal cysteines), of 3_ASIC_FP or 4_ASIC_FP concatemers were isolated from control and BMOE-treated oocytes and analyzed under reducing conditions (Fig 6A and 6B). In the absence of BMOE ASIC1a wt and G433C migrate essentially as monomers while treatment at the cell surface with BMOE stabilizes ASIC1a oligomers that appear as three main bands corresponding to a monomer, a dimer and a tetramer (Fig 6A). In control oocytes expressing 3_ASIC_FP, ASIC1a is detected as 3 bands corresponding to a monomer (I), a dimer (II) and a trimer (III) (Fig 6B). Crosslinking the 3_ASIC_FP at the cell surface identifies ASIC1a oligomers resolved mainly as band I, II and IV oligomers. A similar migration pattern was observed for 4_ASIC_FP expressed at the cell surface. The full-length 4_ASIC_FP protein at the cell surface could not be consistently detected in control oocytes, however in the BMOE-treated counterparts, band IV was present. We could not detect ASIC1a complexes migrating as bands with higher MW than band IV. We verified our observations in CHO cells that also express functional ASIC1a (S2 Fig). The migration pattern of band I to IV oligomers in CHO cells was similar to that in oocytes. Analysis of the ASIC1a channel complex at the surface of cells expressing ASIC1a wt, G433C, the 3_ASIC_FP, and 4_ASIC_FP fusion proteins clearly identifies, for all ASIC1a constructs, complexes that migrate as a tetramer after crosslinking with BMOE (Fig 6C). Fig 6D illustrates the relative abundance of the four different oligomeric forms (bands I to IV) of ASIC1a (wt), G433C (433), 3_ASIC_FP (3xFP), and 4_ASIC_FP (4xFP) in oocytes and CHO cells. Without BMOE (left panel), the ASIC1a wt, or G433C migrate essentially as monomers (band I). In cells expressing the 3_ASIC_FP, ASIC1a migrates mainly as a trimer (band III), but also as dimers and monomers; in cells expressing the 4_ASIC_FP, ASIC1a is found in equal amount as tetramers (band IV), trimers dimers and monomers. After treatment of ASIC1a wt with BMOE, the abundance of the dimers, trimers and tetramers increases; for the BMOE-treated G433C, the tetramer becomes the most abundant oligomeric form. In cells expressing the 3_ASIC_FP, the most abundant oligomer stabilized with BMOE is the tetramer, and not anymore the trimer as in the absence of BMOE. This suggests that the 3_ASIC_FP is complemented with a single subunit to form a tetramer at the cell surface. In cells expressing the 4_ASIC_FP a slight increase in band IV corresponding to the tetramer was observed. Our experiments show that the ASIC1a oligomer with a size corresponding to that of a tetramer, is stabilized by BMOE at the surface of both 3_ASIC_FP and 4_ASIC_FP expressing cells.

**Fig 7:**
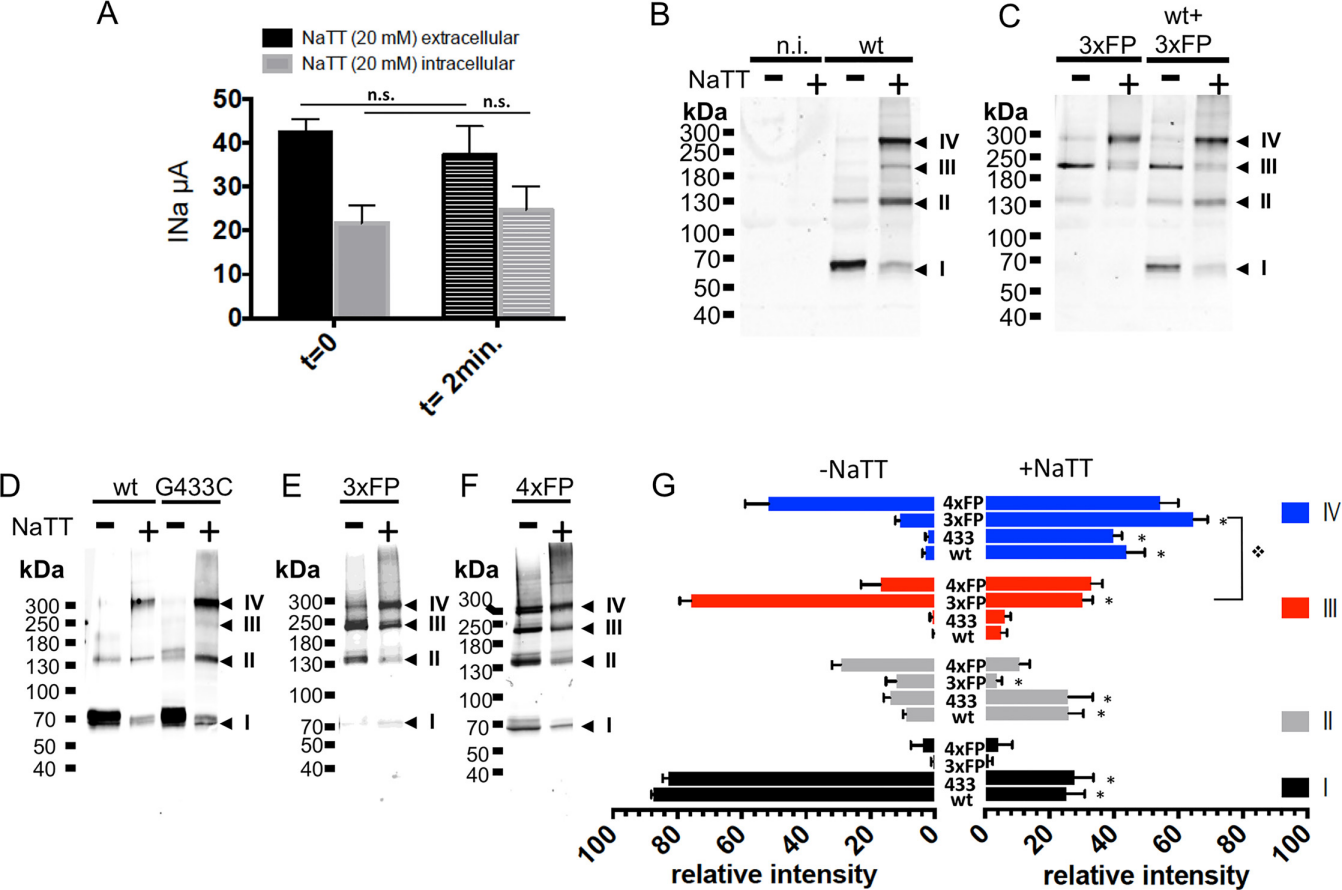
Oligomeric states of affinity-purified ASIC1a isolated from cells expressing functional ASIC1a trimeric and tetrameric concatemers, under oxidizing conditions. A: Effects of extracellular (black bars) or intracellular (grey bars) perfusion of NaTT (20 mM) on ASIC1a currents measured in Xenopus oocytes before (t = 0) and after perfusion (t = 2min.). Bars represents means ± SE (n = 28). B-C: Anti-ASIC1a western blots of control or NaTT-treated (0.3 mM NaTT) affinity-purified fractions from Xenopus oocytes, non-injected (n.i.), or expressing ASIC1a (B), or 3xFP alone or co-expressed with wt ASIC1a (C). D-F: Anti-ASIC1a western blots of affinity-purified fractions from ASIC1a in CHO cells expressing either ASIC1a monomers wt or G433C mutant (D), 3xFP (E), 4xFP (F) and treated with NaTT (0.3 mM). G: Relative intensities (mean ±SD) of each of the 4 bands (I to IV) corresponding to ASIC1a oligomers identified on SDS-PAGE from cells (Xenopus oocytes and CHO cells) expressing ASIC1a wt (n = 9), G433C (n = 5), 3xFP (n = 7), or 4xFP (n = 4), without or after treatment with 0.3 mM NaTT. * denotes p<0.01

The homotetrameric ASIC1a complexes detected at the cell surface for the different ASIC1a constructs could potentially result from an aberrant assembly state induced by the crosslinker BMOE. To test this possibility, we used sodium tetrathionate (NaTT) as an alternative approach to stabilize the intersubunit interactions by favoring the formation of disulfide bonds between cysteines. As shown in Fig 7A, NaTT at concentrations up to 20 mM applied either intracellularly or externally did not affect ASIC1a activity. Western blot analysis performed under non-reducing conditions (Fig 7B) shows that NaTT stabilizes ASIC1a wt oligomers essentially as dimers and tetramers (bands II and IV). In oocytes expressing a functional ASIC1a current (7.0±0.94 μA, n = 16) the 3_ASIC_FP migrates essentially as band III under reducing conditions; however treatment with 0.3 mM NaTT shifts the 3_ASIC_FP oligotrimer to a tetramer (band IV) that becomes the main ASIC1a oligomer (Fig 7C). Coexpression of ASIC1a wt and 3_ASIC_FP increases the ASIC1a current by 2 fold (14.1±1.8 μA, n = 16). This increase in ASIC1a current best correlates with an increase in the ASIC1a tetramer: compared with the condition of expression 3_ASIC_FP alone, the abundance of the ASIC1a tetramer (when normalized to that of the trimer in the absence of NaTT, Fig 7C), increases by a factor of 2.1±0.4 (n = 3 independent blots) with the coexpression of ASIC1a wt, whereas both the ASIC1a monomer and trimer remain as minor bands.

In CHO cells expressing ASIC1a wt, G433C, 3_ASIC_FP, or 4_ASIC_FP, the migration pattern of the ASIC1a oligomers obtained after NaTT treatment (Fig 7D–7F) was similar as that generated by crosslinking with BMOE (Fig 6C): the 3_ASIC_FP protein detected mainly as a trimer in controls, migrates predominantly as a tetramer after treatment with 0.3 mM NaTT (Fig 7E). The 4A_SIC_FP that is detected as a tetramer in controls did not increase significantly after NaTT treatment. No oligomers with higher MW than a tetramer could be reproducibly detected in cells expressing 4_ASIC_FP (Fig 7F) indicating that the 4_ASIC_FP is not complemented to form a higher order oligomer. Fig 7G summarizes the results described above and highlights the statistical significance of the changes in relative band intensities resulting from NaTT treatment for each of the tested ASIC1 forms. Like BMOE (see Fig 6D), NaTT stabilizes the band IV oligomer made of a 3_ASIC_FP protein. For all the constructs expressed in CHO and oocytes (ASIC1a, wt, G433C, the 3_ASIC_FP or 4_ASIC_FP), the major ASIC1a complex migrates as a tetramer after NaTT treatment. These experiments reproduce the observations made with the trimeric and tetrameric concatemers in Fig 6 and confirm that upon expression of both 3_ASIC_FP or 4_ASIC_FP concatemers, the tetrameric oligomer of ASIC1a is the most abundant form obtained after stabilization of the channel complex with either BMOE at the cell surface or NaTT.

## Discussion

This work addresses the state of oligomerization of the ASIC1a complex *in situ*, analyzed by Western blotting after stabilization of the intersubunit interactions. Using different ASIC1a constructs including mutants with additional or substituted cysteines, we could show that the crosslinked ASIC1a channel complex is resolved by SDS-PAGE consistently as four distinct bands with differences in their relative intensities. Comparative analysis of their migration on SDS-gel with ASIC1a concatemers, showed that these oligomers represent respectively monomers, homodimers, -trimers, and -tetramers of ASIC1a. All the ASIC1a cDNA constructs expressed in our cell expression systems were functional. The expression ASIC1a wt, G433C, trimeric or tetrameric concatemers consistently resulted in the assembly of a homotetramer as the most predominant ASIC1a oligomer when stabilized with BMOE at the cell surface or with NaTT on the affinity-purified proteins. Our experiments show that the trimeric ASIC1a fusion protein is complemented by an ASIC1a monomer, to form a tetrameric channel complex, which it is not the case for the tetrameric ASIC1a fusion protein. Thus, our experiments reproducibly identify a major ASIC1a tetramer *in situ* in cell that express functional ASIC1 channels

The functional ASIC1a channel expressed in oocytes or CHO cells are resolved by SDS-PAGE as a monomer and three additional distinct oligomers. Regarding the nature of these oligomers, we have provided strong evidence that these oligomers are homomultimers of ASIC1a. The resolution of the two highest MW oligomers (bands III and IV) depended on the availability of cysteines for crosslinking in the TM1 and in the channel vestibule, where ASIC1a subunits are in close contact. Furthermore, the apparent size of these oligomers matches perfectly that of fusion proteins made of dimers, trimers and tetramers of ASIC1a subunits. We can thus safely conclude that the four ASIC1a bands resolved on SDS-gels represent the stabilized forms of monomeric, dimeric, trimeric, and tetrameric ASIC1a oligomers.

These four ASIC1a oligomers may originate from the dissociation of one or more non-crosslinked subunits from the native ASIC1a complex. Alternatively, ASIC1a channels might assemble and migrate to the surface as distinct oligomers. Our experiments cannot conclusively discriminate between these two possibilities, but seem to favor the first hypothesis. For instance, the abundance of the tetramer relative to the other oligomers obtained from ASIC1a stabilized at the cell surface with BMOE, differs between the G433C and the G433C-ΔC_Ct_ mutant lacking the cysteines in the C-terminus (compare Figs 6D and 4B respectively): the tetramer (band IV) was the most important oligomer resolved with the G433C and represented 60.8 ± 5.8% of all the oligomers, whereas the tetramer represented only 14.0 ± 6.1% for the G433C-ΔC_Ct_; for both constructs the relative abundance of the trimer (band III) was similar, but that of the monomer (band I) and the dimer (band II) was significantly lower for the G433C (8.2 ± 2.8% and 15.7±5.3% respectively) than for the G433C-ΔC_Ct_ (36.1± 7.8% and 30.7±4.3% respectively). Thus the relative importance of the tetramer over the other oligomers identified on SDS-gel depends on the presence of cysteines in the C-terminus of ASIC1. We know from our data (see Fig 1) and from the work published by Zha et al. [14] that the cysteines in the C-terminus are involved in disulfide bonds and stabilize subunits interactions. This suggests the availability of the cysteines in the C-terminus determines the efficiency with which BMOE stabilizes the ASIC1a complex and the ability of unlinked subunits to dissociate from the tetrameric but not from the trimeric complex. As additional argument, we observed differences in the capacity of NaTT and BMOE to stabilize the ASIC1a tetramer resolved by SDS-PAGE. As shown in Figs 6D and 7G, BMOE is relatively inefficient to stabilize ASIC1a wt as a tetramer (17.8 ± 4.5%, n = 17), whereas for ASIC1a wt stabilized with NaTT, the major oligomer resolved on SDS-gel migrates as a tetramer (44 ± 5.6%, n = 9, p<0.01). This difference between NaTT and BMOE in their efficiency to stabilize the ASIC1a tetramer is not surprising because of their different mode of crosslinking cysteine residues. The short treatment period (5 min) of cells with BMOE makes it unlikely that the reagent affects the subunit assembly of the ASIC1a complex during its biosynthesis. Taken together our experiments favor the hypothesis that the ASIC1a trimer originates essentially from the dissociation of a single subunit from an incompletely stabilized ASIC1a tetramer.

It remains possible that the four main oligomers resolved on SDS gels represent unnatural misassembled channels resulting from BMOE treatment. We observed however the same four oligomers in non-reducing western blots of affinity-purified ASIC1a wt or G433C samples that had been treated with NaTT. Under these conditions, the most predominant oligomer resolved on SDS-gel was the tetramer, and the trimer represented at best a minor band. This observation makes the possibility of a misassembled ASIC1a channel complex more theoretical than factual.

Further evidence pointing to the importance of the tetrameric over the trimeric ASIC1a assembly is the observation that the trimeric ASIC1a fusion protein is complemented by a subunit to form a tetramer at the cell surface. Western blots of trimeric and tetrameric ASIC1a fusion proteins (3_ASIC_FP, 4_ASIC_FP) reveal bands with the expected MW for the fusion proteins, but also detect forms with MW that correspond to multiples of ASIC1a subunits (blots in Figs 5–7). As mentioned previously, this could be due to an incomplete biosynthesis or to a proteolytic cleavage of the ASIC1a fusion proteins. Upon expression of the 3_ASIC_FP in Xenopus oocytes and in CHO cells, the ASIC1a is resolved by SDS-PAGE, under reducing conditions and in the absence of BMOE, as a major (≥60%) trimeric ASIC1a protein. After oxidation with NaTT or crosslinking with BMOE, ASIC1a migrates as a predominant tetramer (≥60%) with a parallel decrease in the relative amount of ASIC1a trimeric protein, when compared to controls. This can be interpreted as a complementation of the trimeric ASIC1a protein with a single subunit to form a tetrameric channel complex at the cell surface. We could not obtain any evidence for a complementation of the ASIC1a tetrameric fusion protein (4_ASIC_FP) with an additional subunit at the cell surface. This strongly suggests that the ASIC1a tetramer is the preferential assembly state at the cell surface and suggests it may represent the relevant oligomeric state for ASIC1a function.

We realize that we do not provide direct evidence that the ASIC1a tetramer, that represents the predominant oligomer in CHO and Xenopus oocytes, is indeed the functional oligomer. We cannot formerly exclude that the ASIC1a trimer is the functional oligomer; however if this would be the case, we have consider that in these cells expressing functional ASIC1a current, there is a prominent miss-assembly of ASIC1a into tetramers at the cell surface. A native ASIC1a tetrameric organization is supported by previous functional studies showing a four subunit stoichiometry for ENaC [7–10, 16]. ENaC is made of 3 homologous subunits, α, β, and γ that share 15–20% identity with ASIC1 and a similar membrane topology [2]. Most of the functional studies on ENaC, including channel interaction with different blockers, analysis of specific mutations, expression of subunit concatemeric constructs, and biochemical studies, concluded that ENaC is made of four subunits, 2α, 1β, and 1γ, arranged pseudosymmetrically around a single channel pore [7, 8].

The available crystal structures of the C-terminally truncated chicken ASIC1 reveal a trimer [3, 4, 6, 17]. Since these studies do not provide either any direct evidence that the crystallized protein represents the functional oligomeric form of the channel, the functional relevance of these ASIC1a structures still remains to be elucidated. For instance, none of the available ASIC1 structures show any density features consistent with the presence of permeant cations deep into the pore. This contrasts with the KcsA K^+^ channels, the prokaryotic Cl^-^ channel, the Ca^2+^ channel Orai, or the ligand-gated GLIC channel that were crystallized in the presence of permeant ions, revealing at the atomic level their interactions with pore-lining residues [18–21]. The crystal structure of cASIC1 has been used to provide a structural rationale for different biophysical properties of ASIC1 channel function such as their activation and desensitization [15, 22, 23]. However, these studies were not designed to specifically address the question of the subunit stoichiometry of ASIC1, and their conclusions cannot be used to refute a tetrameric organization of the native ASIC1 channel. Atomic force microscopy (AFM) imaging made on purified ENaC and ASIC1 channel complexes bound to specific antibodies revealed angles between the Fab fragments that were consistent with trimeric channels [24, 25]. Such imaging technique critically lacks spatial resolution for the determination of membrane protein structure and the trimeric assembly ASIC1 or ENaC channels was not verified biochemically. Finally a single-molecule photobleaching approach of fluorescently-tagged ASIC1 and ASIC2 channels expressed in Xenopus *laevis* oocytes was used recently to determine the channel stoichiometry by counting the bleaching steps obtained with fluorescent spots at the cell surface, as indicator of the number of subunits in the channel complex. The majority (62%) of fluorescent spots bleached in two or three steps that were considered as reflecting a trimeric stoichiometry of ASIC channels. Limitations of this approach include the stability of these GFP-tagged ASIC fusion proteins during biosynthesis, and the potential effect of the fluorophore on channel assembly and activity.

Our data do not question the fact that cASIC1, fused to GFP and carrying a truncation of its C-terminus, assembles and crystallizes as a trimer when expressed in insect Sf9 cells [3, 4, 6, 17]. Simply they raise a fundamental question that still needs to be carefully addressed: does the trimeric crystal structure represent the functional ASIC1a channel *in situ*, in its membrane environment? Further studies are needed to determine whether detergents, deletions in the C-tail, fusions with GFP, or overexpression conditions, may alter the ASIC1a assembly during biosynthesis. Our results certainly will not provide the last words on the subunit stoichiometry, but hopefully will promote new studies to solve the discrepancy between the crystal structure of ASIC1 and the biochemical analysis of the ASIC1a channel complex *in situ*.

## Materials and Methods

### Cloning and site-directed mutagenesis

The coding sequence of human ASIC1a was cloned in the pSDEasy vector. An octahistidine coding sequence (H_8_) was introduced in frame using XhoI and SalI restriction sites (H_8_-ASIC1a.psd). This insert provides as well a BmgBI following the octahistidine-encoding sequence in frame with and just preceding the SalI site. All subsequent constructs were based on this vector.

The ASIC1a construct lacking the cysteines in the C-terminus, H_8_-hASIC1a-C466A/C471A/C497A/C528Stop (ASIC1a-ΔC_Ct_) was used as template for generating the C59V, C61S, C59V/C61S, V74C, Y426C, G430C, G433C cysteine substitutions (QuickChange, Stratagene). Mutations were verified by sequencing (Synergene Biotech, Zurich, Switzerland).

In order to generate ASIC1a concatemeric fusion proteins, a PspXI site was introduced by PCR at the 3’ end of the H_8_-hASIC1a coding sequence, between the codon encoding the terminal Cys residue, that was conserved, and the stop codon (ASIC-PspXI-Stop). A linker sequence encoding one Asn followed by seven Gln residues (N_1_Q_7_) was cloned into the PspXI site in such a way that one single PspXI site was regenerated at the distal cloning site (ASIC-N_1_Q_7_-PspXI-Stop). This linker is similar to the octa-Gln used to generate concatemeric ENaC constructs [7]. The dimeric ASIC (H_8_-hA1a-N_1_Q_7_-hA1a.psd) was generated by subcloning the BmgBI-PspXI insert of H_8_-ASIC-N_1_Q_7_-PspXI-Stop into the BmgBI-SalI linearized H_8_-ASIC1a.psd plasmid. This cloning eliminates the SalI site of the receiving plasmid and thus allows maintenance of a single SalI site, corresponding to that present in the new insert. The same strategy was used to generate, successively, the trimeric (H_8_-(hA1a-N_1_Q_7_)_2_-hA1a.psd) and the tetrameric (H_8_-(hA1a-N_1_Q_7_)_3_-hA1a.psd) forms using H_8_-hA1a-N_1_Q_7_-hA1a.psd and the resulting H_8_-(hA1a-N_1_Q_7_)_2_-hA1a.psd as receiving plasmids, respectively.

For expression in CHO cells, the XhoI-XbaI fragments encoding the His-tagged ASIC constructs were subcloned into pCDNA3.1(+)_zeo (Life Technologies, Zug, Switzerland).

### Expression in Xenopus oocytes and in CHO cells

Complementary RNAs were synthesized *in vitro* with SP6 RNA-polymerase (Promega, Dübendorf, Switzerland) from H_8_-ASIC1a cDNA encoding vectors previously linearized with FspI. Healthy stage V and VI *Xenopus laevis* oocytes were pressure-injected with 10 ng of cRNA.

CHO-K1 cells were plated on P100 dishes and transfected with 15 μg plasmid DNA per plate using Lipofectamine-2000 (Life-Technologies), according to the manufacturer instructions. In experiments involving ASIC1a concatemers, the amount of vector for the latter was adjusted taking into account the increase in plasmid size: 18, 20, and 22 μg DNA for the dimeric, trimeric, and tetrameric construct, respectively. Plasmid dilutions where supplemented with shredded ultra-pure herring sperm DNA (Invitrogen # 15634–017) as to obtain the same amount of total DNA in each transfection, and the amount of Lipofectamine-2000 was adjusted accordingly. After 8–16 h incubation with the transfection mix, fresh medium (DMEM:F12, 3.6% FCS, 1% PenStrep) was added and cells were further incubated for ~24h, in experiments concerning monomeric ASIC only, and up to 60 h when concatemeric ASIC forms were also involved.

### Electrophysiology

Electrophysiological measurements were performed 24–48 h after oocyte injection with ASIC cRNA. Macroscopic ASIC1a currents were elicited every 40 s by rapid changes in extracellular pH from 7.4 to 6.0, and were measured using either the two-electrode voltage-clamp for whole-cell currents or the cut-open oocyte technique when intracellular perfusion was needed, as previously described [26]. For the two-electrode voltage clamp experiments, reagents were directly added to the extracellular solutions: 10, 100, and 1000 μM Cd^2+^, or 2 mM BMOE. In experiments investigating the effects of intracellular BMOE on ASIC1a activity, 100nl of a 10mM BMOE or vehicle (DMSO) solution were injected per oocyte 60 to 180min before electrophysiological measurements. In the cut-open oocyte experiments, the voltage clamp was performed using a Dagan cut-open oocyte voltage clamp apparatus (Dagan Corporation, Minneapolis, MN; model CA-1 high performance oocyte clamp). 24 hours after RNA injection, oocytes were perfused extracellularly and intracellularly. Extracellular solutions contained, in mM: 80 Na-gluconate, 10 HEPES, 10 TEA-Cl, 5 BaCl_2_, 1 MgCl_2_ and 0.5 CaCl_2_, pH 7.5/6.0 adjusted with NMDG. The intracellular side of oocytes was perfused either using a control solution (in mM: 90 K-gluconate, 10 HEPES, 10 KCl, 2 Na-gluconate, 1 MgCl_2_, 0.2 BAPTA, pH 7.35 adjust with NMDG) or a solution supplemented with 2mM of BMOE. The BMOE perfusion lasts 6 minutes. Oocytes were then washed intracellularly with the control solution.

Electrophysiological measurements with transfected CHO cells was carried out as described elsewhere (ref Alijevic and Kellenberger 2012).

### Biochemistry

#### Cell-surface crosslinking and biotinylation

Oocytes expressing ASIC1a were incubated in 1 ml MBS solution supplemented with 2 mM BMOE (5 and 120 min at 19°C). Oocytes were then washed three times and then labeled (15 min incubation) on ice in 1 ml of biotinylation buffer (in mM: 10 Triethanolamine, 150 NaCl and 2 CaCl_2_) supplemented with 1 mg/ml biotinylation reagent. Oocytes were incubated for 5 min in 1 ml of quenching buffer (in mM: 192 Glycine, 25 Tris-HCl at pH 7.5, in MBS solution) and subsequently washed 3 times with MBS. Oocytes were lysed in a streptavidin binding buffer (SBB, 20 μl LB/oocyte) containing 1% (v/v) Triton and, in mM: 5 EDTA, 100 NaCl, 40 Tris-HCl (pH 7.5), and protease inhibitor cocktail.

Transfected CHO cells were washed with PBS containing 0.1 mM CaCl_2_ and 1 mM MgCl_2_ (PBS-CM) and then treated for 5 min at 22°C in the presence of 4 ml PBS-CM containing 2 mM BMOE or vehicle (DMSO, 2% final). After washing with chilled PBS-CM, plates were placed on ice in a cold room and cells were incubated for 15 min in the presence of 4 ml PBS-CM supplemented with 0.25 mg/ml of biotinylation reagent. The reaction was quenched by replacing the biotinylation solution with 10 ml of 100 mM glycine in PBS-CM and further incubation for 20 min in the cold. Lysates were prepared by scraping cells in 3 ml membrane isolation buffer (in mM): 50 Tris/HCl (pH 7.0 at RT), 150 NaCl, 5 MgCl_2_, 1 DTT, and protease inhibitor cocktail, snap-frozen in liquid nitrogen, and stored at -70°C. After thawing, raw lysates were incubated for 30 min on an orbital shaker at 4°C in the presence of 0.1 mg/ml DNaseI (Roche Diagnostics AG, Rotkreuz, Switzerland). The membrane pellet obtained after 30 min centrifugation at 20,000g (4°C) was resuspended in SBB and proteins were solubilized by 45 min incubation on an orbital shaker at 4°C, and centrifuged for 12 min as before.

#### Affinity purification of biotinylated fractions on streptavidin beads

The Triton-soluble fractions from biotinylated oocytes or transfected CHO cells were incubated for 1 h at 22°C with 25 μl (bed volume) of streptavidin-beads. Beads were recovered by 2 min centrifugation at ~1,000g and washed three times in SBB. Bound proteins were then eluted by resuspending the drained beads in 40 μl of sample buffer (25 mM DTT, final concentration) and heating at 95°C for 5 min. Unbound proteins were then recovered by centrifuging through mini-filters (EVE-208, Evergreen Scientific, Los Angeles, CA). Pilot studies using lysates from non-biotinylated oocytes and cells were carried out to assess the absence of non-specific binding of His-tagged hASIC1a to streptavidin (not shown). In certain experiments, after two washing steps, the streptavidin beads were incubated on an orbital shaker for 20 min at 4°C in the presence of either 1 mM BMOE or vehicle. Reagent was removed by thorough washing before elution with sample buffer.

#### Pull-Down with nickel-NTA-Agarose beads of extracts from oocytes and from transfected CHO cells

Cell membranes were isolated from either oocytes or transiently transfected CHO cells as described before and solubilized in Nickel binding buffer (NBB) containing 1% Triton and, in mM: 150 NaCl, 50 Tris-HCl, at pH 7.5, 20 Imidazole, 1 DTT, and the same protease inhibitor cocktail as that described for oocytes extract preparation. After 45 min incubation on an orbital shaker at 4°C, lysates were centrifuged for 12 min at 20,000g, and the soluble phase was subjected to batch affinity chromatography on Ni-NTA-agarose beads by incubating 3 h at 4°C on an orbital shaker. Beads were washed three times in NBB. In those experiments in which oxidation of bound proteins with NaTT was going to be tested, two washes with X-link buffer (1% Triton and, in mM: 100 NaCl, 50 Tris-HCl, pH 7.5) followed. The thus washed beads were then rotated for 20 min at 4°C in X-link buffer supplemented or not with 0.3 mM NaTT. Non-oxidized cysteines were blocked by washing beads in X-link buffer supplemented with 30 mM N-ethylmaleimide. Bound proteins were subsequently eluted by heating beads with Sample Buffer supplemented with 30 mM of each N-ethylmaleimide and EDTA. Alternatively, beads were rotated as described before in X-link buffer supplemented or not with 1 mM BMOE. Bound proteins were subsequently eluted by heating beads with Sample Buffer supplemented with 30 mM of each N-ethylmaleimide and DTT.

#### Western-Blot, Infrared detection

Proteins were resolved by SDS-PAGE on 5–15% acrylamide gradient minigels under reducing conditions, except for samples from oxidation experiments, for 1 h at 200V along with pre-stained molecular weight markers. Proteins were then electrotransferred onto nitrocellulose (Whatman Protran #10401396) for 3h at 100V. After 1 h of blocking at RT in 0.1% (w/v) casein solution, the membrane was incubated overnight with the primary antibody in 1% milk-TBS-Tween. After three rounds of washing over 20–30 minutes in TBS-Tween, the blot was incubated 1 h in the presence of IRDye-conjugated secondary antibodies diluted 1/12,000 in 0.1% casein solution. After washing, the blot was scanned with an Odyssey Infrared Imaging System (LI-COR Biosciences). In the images, lanes from the same blot are shown along with their corresponding molecular weight marker. When indicated, they have been cropped together to display relevant data. Because the expression levels differed largely, especially at the cell surface, e.g. between monomeric and certain concatemeric forms, the intensity of the N-IR images of the corresponding lanes was adjusted as to render visible the lanes with the faintest bands without saturating that of the most strongly expressed proteins. Lanes corresponding to treated samples and their corresponding controls are analyzed in all cases with the same intensity.

#### Quantitative analysis of apparent molecular weights and intensities of western blot bands

To determine the apparent molecular weight of each band, we analyzed the raw scans with the ImageJ software (Rasband, W.S., ImageJ, U. S. National Institutes of Health, Bethesda, Maryland, USA, http://imagej.nih.gov/ij/, 1997–2014) in order to measure the distances migrated by the molecular weight marker’s bands. Those distances were plotted against the respective theoretical weight of each band and fitted it to an exponential curve in an Excel sheet (Microsoft). The function of the corresponding curve was used to calculate in each blot the apparent molecular weight value of the ASIC1a-immunostained bands on the basis of their respective migration distances. This protocol was repeated for each gel and lane. We have verified that the molecular weight estimated using this protocol was reproducible and identical with the two different molecular weight markers used in this study (see S1 Fig). Finally, the mean (± SD) of the estimated apparent molecular weights was calculated and reported for each ASIC1a construct and crosslinking condition. The intensities of bands were analyzed using the Odyssey 2.1 software. For samples obtained after crosslinking experiments, the intensities of the main bands comprising 95% or more of the total lane intensity (after baseline correction) were added and the relative intensity of each band was calculated. In certain blots, certain bands appeared to migrate as doublets, with a difference in apparent molecular weight of ca. 10%. In such cases, these doublet bands were considered as a single species and their intensities were hence added together. Finally, the average of the ratios measured for each band in individual experiments was calculated and reported for each of the ASIC1a constructs (± SD).

### Statistical analysis

Statistical significance of differences between samples or conditions was analyzed by unpaired t-tests; the calculated p values are indicated throughout the text or in the corresponding figure legends.

### Chemicals and Cell lines

Bis(maleimido)ethane (BMOE #22323), the biotinylation reagent EZ-link sulfo-NHS-SS-biotin (#21331) and immunopure immobilized streptavidin gel (#20349) were purchased from Thermo Scientific (USA). Anti-His-tag monoclonal antibody was from GE Healthcare (#27-4710-01). Polyclonal anti-ASIC1 (#ASC-014) was purchased from Alomone Labs (Jerusalem, Israel). Polyclonal goat antibody anti-rabbit IRDye 800CW (#926–32211), IRDye 680CW (#926–32221), and goat anti-mouse IRDye 680CW (#926–68070) were from Li-Cor Biosciences GmbH (Bad Homburg, Germany). Pre-stained protein molecular weight ladders were from PeqLab Biotechnologie (peqGold GmbH, Erlangen, Germany, Protein Marker, #27–2210, range 10–250 kDa) or from Thermo Scientific (Spectra Multicolor High Range Protein Ladder, #26625, range 40–300 kDa). CHO-K1 cells have been purchased from the American Type Culture Collection (ATCC, CCL-61).

## Supporting Information

S1 Fig Estimation of the mass of the ASIC1a oligomers using two different protein molecular weight markers. A. Anti-His-tag Western blot of affinity-purified fractions from oocytes, non-injected or expressing either wt ASIC1a (ASIC1a), ASIC1a-ΔC_Ct_, or ASIC1a-G433C (G433C) and treated before elution from the Ni^2+^-NTA-agarose beads with BMOE (see methods). B. Correlation between the MW of the four oligomers detected in blots, estimated with either of the two commercial MW markers run together in each of the gels and ranging up to 250 kDa (peqGold, peqlab) or to 300 kDa (Spectra, Thermo). A slope of 1.00 (95% confidence interval 0.9879 to 1.012) was calculated by linear regression analysis of the plot. Symbols represent means±SD.
(TIF)

S2 Fig Recordings of ASIC1a currents elicited at pH 5 in CHO cells expressing ASIC1a wt and G433C. ASIC1a were recorded in whole-cell patch configuration.
(TIF)
