# Supplementary figures and images for: The Human Acid-Sensing Ion Channel ASIC1a: Evidence for a Homotetrameric Assembly State at the Cell Surface
Source: PLoS One. 2015 Aug 7;10(8):e0135191. doi: 10.1371/journal.pone.0135191 (PMC4529235; doi:10.1371/journal.pone.0135191)

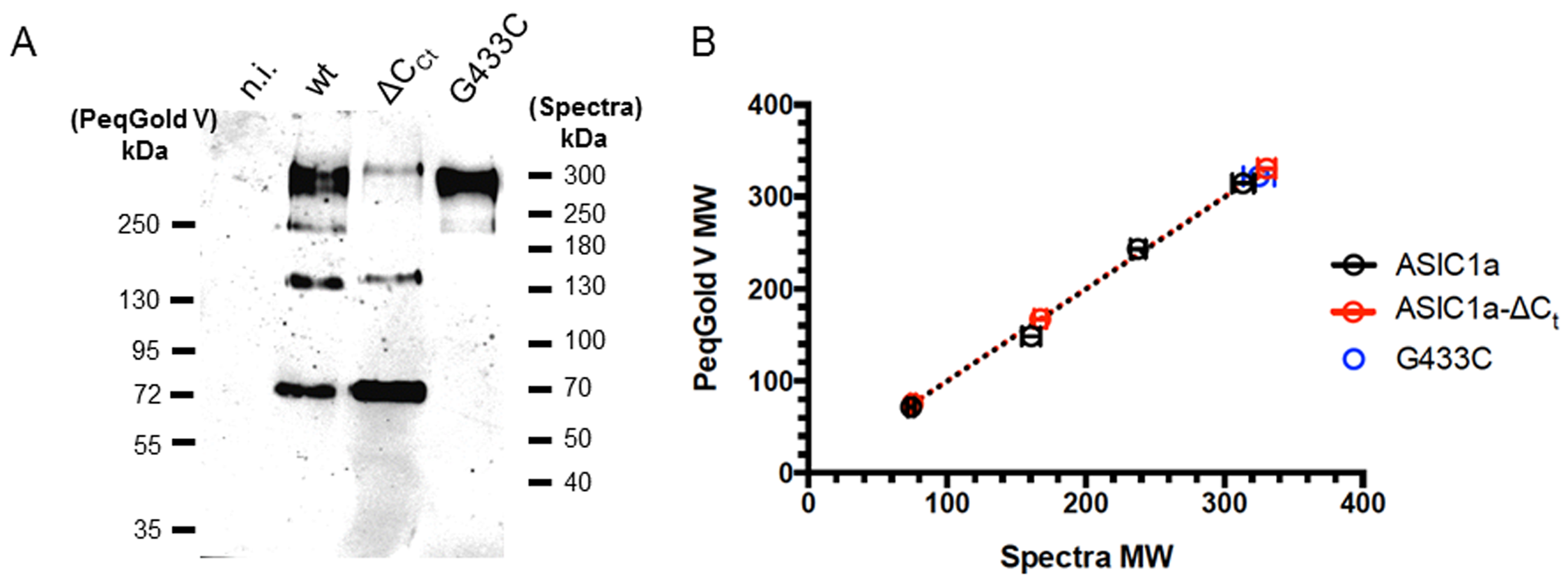

Supplement: S1 Fig — A. Anti-His-tag Western blot of affinity-purified fractions from oocytes, non-injected or expressing either wt ASIC1a (ASIC1a), ASIC1a-ΔCCt, or ASIC1a-G433C (G433C) and treated before elution from the Ni2+-NTA-agarose beads with BMOE (see methods). B. Correlation between the MW of the four oligomers detected in blots, estimated with either of the two commercial MW markers run together in each of the gels and ranging up to 250 kDa (peqGold, peqlab) or to 300 kDa (Spectra, Thermo). A slope of 1.00 (95% confidence interval 0.9879 to 1.012) was calculated by linear regression analysis of the plot. Symbols represent means±SD. (TIF) [file pone.0135191.s001.tif]

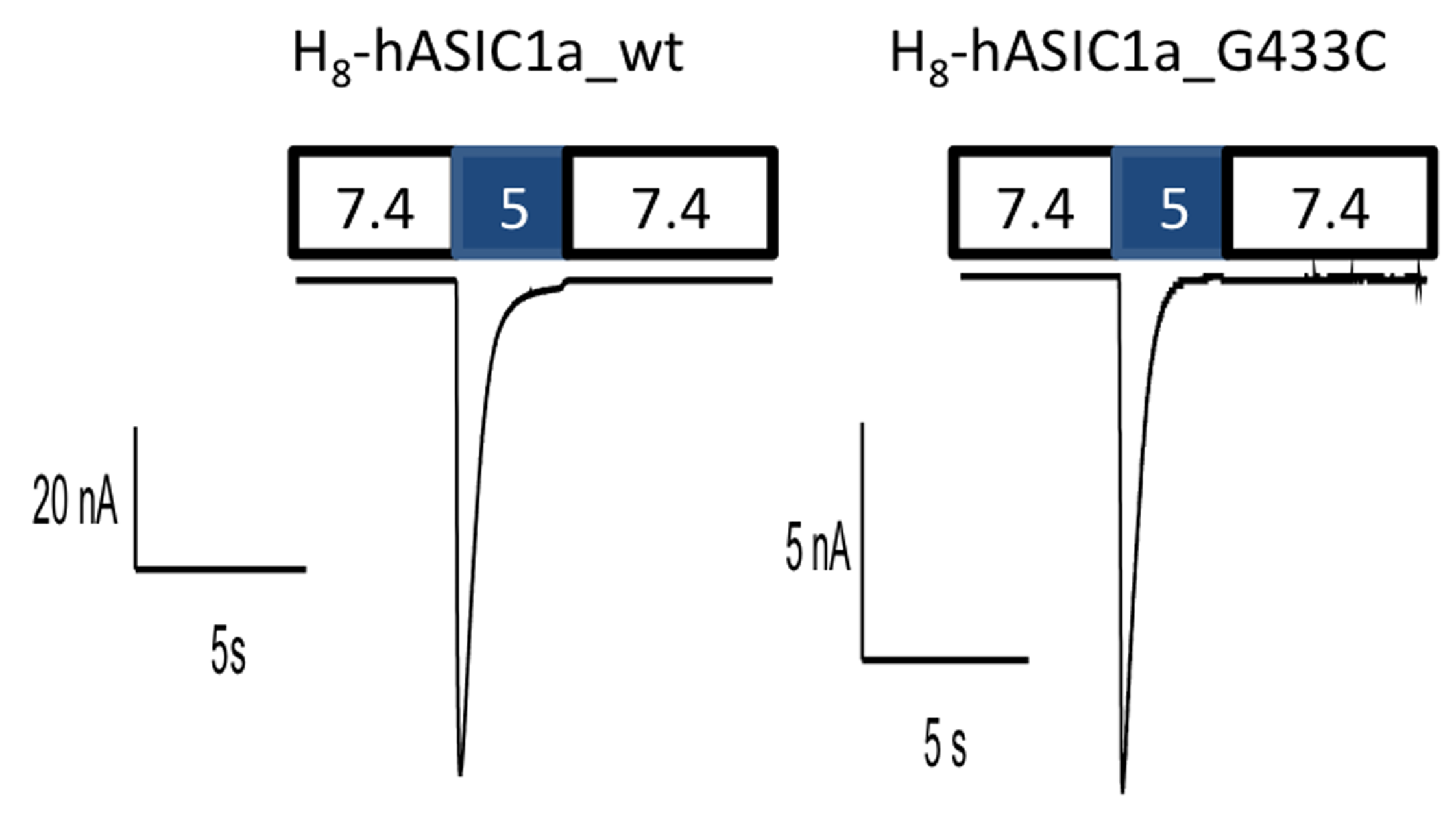

Supplement: S2 Fig — ASIC1a were recorded in whole-cell patch configuration. (TIF) [file pone.0135191.s002.tif]
